# Potential drug interactions in drug therapy prescribed for older adults at hospital discharge: cross-sectional study

**DOI:** 10.1590/1516-3180.2019.013405072019

**Published:** 2019-10-31

**Authors:** Bianca Menezes Dias, Fabiana Silvestre dos Santos, Adriano Max Moreira Reis

**Affiliations:** I Pharmacist, School of Pharmacy, Universidade Federal de Minas Gerais (UFMG), Belo Horizonte (MG), Brazil.; II MSc. Pharmacist, School of Pharmacy, Universidade Federal de Minas Gerais (UFMG), Belo Horizonte (MG), Brazil.; III PhD. Associate Professor, Department of Pharmaceutical Products, School of Pharmacy, Universidade Federal de Minas Gerais (UFMG), Belo Horizonte (MG), Brazil.

**Keywords:** Drug interactions, Frail elderly, Patient discharge, Drug therapy

## Abstract

**BACKGROUND:**

Older adults with a range of comorbidities are often prescribed multiple medications, which favors drug interactions.

**OBJECTIVES:**

To establish the frequency of potential drug interactions in prescriptions at hospital discharge among older adults and to identify the associated factors.

**DESIGN AND SETTING:**

Cross-sectional study conducted in a public hospital.

**METHODS:**

An initial face-to-face interview, data collection from the electronic medical records (covering sociodemographic, clinical, functional and drug therapy-related variables) and telephone follow-up after discharge were conducted to confirm the medication prescribed at discharge. Drug interactions were identified through the Micromedex DrugReax software, along with interactions that should be avoided among the elderly, as per the 2015 American Geriatric Society/Beers criteria. Multivariable logistic regression was performed.

**RESULTS:**

Potential for drug interactions was identified in the discharge drug therapy of 67.8% of the 255 older adults evaluated (n = 172), and 54.5% (n = 145) of the drug interactions were major. Among the drug interactions that should be avoided among older adults, those that increase the risk of falls were the most frequent. The drug interactions thus identified were independently associated with polypharmacy (odds ratio, OR = 12.62; 95% confidence interval, CI 6.25-25.50; P = 0.00), diabetes mellitus (OR = 2.16; 95% CI 1.05-4.44; P = 0.04), hypothyroidism (OR = 7.29; 95% CI 2.03-26.10; P = 0.00), chronic kidney disease (OR = 3.41; 95% CI 1.09-10.64; P = 0.03) and hospitalization in geriatric units (OR = 0.45; 95% CI 0.22-0.89; P = 0.02).

**CONCLUSION:**

The frequency of potential drug interactions in drug therapy prescribed at discharge for these older adults was high. Polypharmacy, diabetes mellitus, hypothyroidism and chronic kidney disease were positively associated with occurrences of drug interactions, while hospitalization in geriatric units showed an inverse association.

## INTRODUCTION

The number of older adults in the world is expanding rapidly as a consequence of increased quality of life and technological advances.^1^^,^^2^ This demographic transition is increasing the prevalence of chronic-degenerative diseases. One of the consequences is higher consumption of medicines, which implies a great challenge for the healthcare system.^3^

Given that use of several drugs is needed to treat the multimorbidity commonly seen in older adults, it can be expected that the prevalence of polypharmacy among older adults and the risk of drug interactions will increase substantially.^4^ Drug interactions are a common cause of adverse drug reactions among older adults, and these include arrhythmias, acute kidney injury and increased risk of falls.^5^^,^^6^^,^^7^ A direct correlation between the number of medications and their interactions and the risk of adverse drug reactions has been shown.^4^^,^^5^^,^^6^^,^^7^ The reasons for this are multifactorial and particularly include age-related changes in the pharmacodynamics and pharmacokinetics of drugs, reduced renal function and lower hepatic clearance.^6^^,^^7^^,^^8^

At hospital discharge, older adults generally present changes to their drug therapy, with higher numbers of prescribed medications, which leads to higher frequency of drug interactions.^8^ Thus, it is essential to know the frequency of these interactions at hospital discharge among older people and their determinants, in order to promote safer use of drugs among older adults.

## OBJECTIVE

The aims of this study were to determine the frequency of potential drug interactions in the drug therapy prescribed at discharge among older adults and to evaluate the factors associated with this outcome.

## METHODS

### Study design and setting

This was a cross-sectional study carried out in a public hospital located in southeastern Brazil that is responsible for attending public servants. The hospital provides medium and high-complexity levels of care.

### Sample

The sample was non-probabilistic, covering older inpatients admitted from April to November 2017. Age ≥ 60 years was adopted to define older adults, as established by the World Health Organization for developing countries.

### Selection criteria

This study included older patients hospitalized in the internal medicine and geriatric units over the period from April 4 to November 1, 2017. All patients who were hospitalized for more than 24 hours during the period of the investigation at the hospital were invited to participate in the research. The exclusion criteria were defined as occurrences of death during hospitalization, hospitalization for more than 60 days, self-discharge from the hospital or loss of contact after discharge.

### Data collection and ethics

The patients were identified through the computerized system for hospitalization management of the hospital and were approached in person, in the hospital. All patients or their legal guardians gave their agreement to participate by signing an informed consent statement. This study was approved by the research ethics committee of Universidade Federal de Minas Gerais under the approval number 1.952.130.

An interview was conducted with patients to collect sociodemographic information. Clinical information was collected by consulting the patients’ electronic medical record. The interviews and data collection were performed by a pharmacist researcher and recorded in the structured form. The pharmacist researcher also made a telephone follow-up within 48 hours after discharge in order to confirm which medications had been prescribed. The medications prescribed were registered on the structured form.

The dependent variable was the presence of potential drug interactions between the drugs prescribed at hospital discharge, using the data collected from the medical records and the confirmations from the telephone interviews. These potential drug interactions were identified using the IBM Micromedex DrugReax software,^9^ considering the medications prescribed at discharge and confirmed during the telephone contact. DrugReax presents adequate sensitivity and specificity for identifying drug interactions within older adults’ drug therapy.^10^

Drug interactions were classified regarding their severity, adopting the DrugReax specifications: contraindicated (when the medications are contraindicated for concomitant use); major (when the interaction may be life-threatening for the patient and requires immediate medical intervention); or moderate (when the interaction may result in exacerbation of the patient’s clinical condition or require a change of therapy).^9^ This investigation did not include the interactions defined by DrugReax as minor (when the interaction may have limited clinical effects without requiring drug therapy changes) or unknown effects (when the level of severity of the interaction is undefined).^9^

The dose of acetylsalicylic acid (ASA) was observed for the purpose of identifying the interactions involving this drug. For specific interactions involving ASA, the clinical management section of the DrugReax software informs whether this occurs with doses used for analgesia and antipyresis (> 300 mg) or doses used for antiplatelet effect (70-300 mg).^9^ Furthermore, the frequency of potential clinically-relevant drug interactions with non-anti-infective agents that should be avoided among the older adults, as per the 2015 American Geriatric Society (AGS)/Beers criteria,^11^ was established.

The demographic, clinical, functional and drug therapy-related independent variables of these older individuals were collected through interviews conducted during their hospitalization. These data were complemented through a search in each patient’s medical records.

Presence of polypharmacy was defined as use of five or more medications. Admission diagnoses were classified in accordance with the International Classification of Diseases, 10^th^ edition (ICD-10).^12^ The patients were assessed using the Charlson Comorbidity Index (CCI).^13^ The complexity of the drug treatment regimen prescribed at discharge was determined by calculating the Medication Regimen Complexity Index (MRCI), using the Brazilian version.^14^ The drug therapy complexity was stratified as high (MRCI value > 16.5) or not high (MRCI ≤ 16.5), as per the MRCI standardization proposed for Brazilian older adults.^15^ The patients’ vulnerability was evaluated by means of the Vulnerable Elders Survey-13 tool (VES-13).^16^

### Statistical analysis

The data collected were double-entered into a dataset elaborated in EpiData 3.1. The statistical analyses were performed using the Statistical Package for the Social Sciences (SPSS) 25.0 software.

Absolute and relative frequencies were determined for dichotomous variables, and numerical variables were described in terms of their mean (with standard deviation, SD) or median (with interquartile range, IQR). The Shapiro-Wilk test was used to assess whether the data presented normal distribution. Numerical variables were dichotomized using the median. Associations between independent variables and occurrences of drug interactions were evaluated using the chi-square test and Fisher’s exact test, with due regard to the premises of each test.

The level of statistical significance of the study was considered to be P < 0.05. Variables with P < 0.20 in univariate analysis were included in the multivariate analysis through the logistic regression model. The forward stepwise method was used to obtain the final model, and variables presenting P < 0.05 were kept in the model. The goodness of fit of the final model was evaluated using the Hosmer-Lemeshow test, such that P > 0.05 was considered to indicate good logistic regression model fit.

## RESULTS

**Figure 1.**
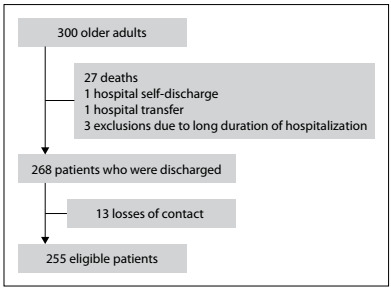
Study flowchart.

In total, 300 older adults were interviewed. Among these, 27 (9%) died during hospitalization, one (0.3%) self-discharged from the hospital, one (0.3%) was transferred to another hospital and three (1%) were excluded due to prolonged hospitalization, since the duration of data collection was limited. Out of the 268 elderly subjects who were discharged, 13 (4.3%) were subsequently lost to contact. Thus, 255 older people were included in this study (Figure 1).

The sample had a median age of 75 years (IQR 13.0) and consisted mostly of women (57.3%). The median score in the Charlson Comorbidity Index was 5.0 (IQR 2.0), and the median score in the Vulnerable Elders Survey-13 (VES-13) was 5.0 (IQR 6.0).

Among the participating older adults, 158 (62.0%) were hospitalized in the internal medicine unit and 97 (38.0%) in the geriatric unit. The median length of hospital stay was 12 days (IQR 10). The most frequent admission diagnoses were respiratory system diseases (64; 25.1%), genitourinary system diseases (43; 16.9%) and circulatory system diseases (30; 11.8%). The most frequent comorbidities among the elderly subjects were arterial hypertension (n = 181; 71.0%), diabetes mellitus (DM) (n = 110; 43.1%) and pneumonia (n = 53; 8%).

The frequency of polypharmacy was 68.2%, and the number of medications used by these older adults was higher after discharge (median = 6.0; IQR 4.0) than the total number of medications used before admission (median = 5.0; IQR 5.0). Potential drug interactions were identified in 173 patients (67.8%), with a median of six interactions per older adult (IQR = 4; minimum = 1, maximum = 15) (Table 1).

**Table 1.** Sociodemographic, clinical and drug therapy-related characteristics of the sample of 255 older adults

Characteristics	Value (%)
Sociodemographic characteristics	
Age in years, median (IQR)	75 (13)
Female gender, n (%)	146 (57.3)
Clinical characteristics	
VES-13 score, median (IQR)	5 (6.0)
Charlson Comorbidity Index, median (IQR)	5 (2.0)
Internal medicine unit, n (%)	158 (62.0)
Geriatrics unit, n (%)	97 (38.0)
Length of hospital stay, median (IQR)	12 (10)
Functional characteristics	
VES-13 score, median (IQR)	5 (6.0)
Admission diagnoses, n (%)	
Respiratory system diseases	64 (25.1)
Genitourinary system diseases	43 (16.9)
Circulatory system diseases	30 (11.8)
Symptoms, signs and abnormal clinical and laboratory findings not elsewhere classified in ICD-10 codes R00-R99	25 (9.8)
Some infectious and parasitic diseases	21 (8.2)
Digestive system diseases	18 (7.1)
Mental and behavioral disorders	14 (5.5)
Endocrine, nutritional and metabolic diseases	11 (4.3)
Other*	40 (15.6)
Comorbidities, n (%)	
Arterial hypertension	181 (70.9)
Diabetes	110 (43.1)
Pneumonia	53 (20.8)
Chronic renal failure	45 (17.6)
Hypothyroidism	43 (16.9)
Heart failure	38 (14.9)
Drug therapy-related characteristics	
Polypharmacy, n (%)	174 (68.2)
Number of medicines used after discharge, median (IQR)	6 (4.0)
Number of medicines used before admission, median (IQR)	5 (5.0)
Patients with interactions, n (%)	173 (67.8)
Number of interactions per patient, median (IQR)	6 (4.0)
Number of interactions per patient, minimum-maximum	1 (15)

ICD-10 = International Classification of Diseases, tenth edition; * = Sum of low-frequency admission diagnoses found in the study but not included in the other categories shown in this table; IQR = interquartile range; VES-13 = Vulnerable Elders Survey 13: individuals with scores ≥ 3 were 4.2 times more likely to be at risk of functional decline within two years, compared with individuals with lower scores.^16^

Overall, 266 types of potential drug interactions were observed, of which 145 (54.5%) were classified as major, 116 (43.6%) as moderate and five (1.9%) as contraindicated. The most frequent major interaction in this study was between amlodipine and simvastatin, which was identified in 16 (6.3%) of all the prescriptions. The ten most frequent major drug interactions and their clinical effects, as per DrugReax, are shown in Table 2.

**Table 2.** Description of major and moderate drug interactions with frequencies higher than four in the drug therapy prescribed at hospital discharge among the 255 elderly subjects, and their clinical effects

Drug interactions	Clinical effects	Absolute frequencies	Relative frequencies (%)
Major			
Amlodipine + simvastatin	Increased risk of myopathy, including rhabdomyolysis	16	6.3
Codeine + ondansetron	Increased risk of serotonergic syndrome	5	2.0
Clopidogrel + omeprazole	Reduced platelet antiaggregant activity	5	2.0
Allopurinol + enalapril	Hypersensitivity reactions	5	2.0
Simvastatin + warfarin	Increased risk of bleeding, including rhabdomyolysis	4	1.6
Simvastatin + diltiazem	Increased risk of myopathy, including rhabdomyolysis	4	1.6
Risperidone + simvastatin	Increased risk of myopathy, including rhabdomyolysis	4	1.6
Donepezil + quetiapine	Increased risk of prolonged QT interval	4	1.6
Clonazepam + tramadol	Increased risk of depression of the CNS	4	1.6
Ciprofloxacin + insulin	Glycemic alterations	4	1.6
Moderate			
Insulin + losartan	Increased risk of hypoglycemia	17	6.7
Insulin + metformin	Increased risk of hypoglycemia	16	6.3
Enalapril + metformin	Increased risk of hypoglycemia	15	5.9
Levothyroxine + omeprazole	Reduced effectiveness of levothyroxine	14	5.5
Enalapril + insulin	Increased risk of hypoglycemia	14	5.5
Levothyroxine + simvastatin	Reduced effectiveness of levothyroxine	12	4.7
Carvedilol + insulin	Glycemic alterations; reduced hypoglycemic symptoms	11	4.3
Atenolol + metformin	Glycemic alterations; reduced hypoglycemic symptoms	11	4.3
Carvedilol + metformin	Glycemic alterations; reduced hypoglycemic symptoms	7	2.7
Captopril + hydrochlorothiazide	Hypotension risk	6	2.3
Atorvastatin + clopidogrel	Reduced effectiveness of clopidogrel	6	2.3
Enalapril + hydrochlorothiazide	Hypotension risk	5	2.0
Enalapril + furosemide	Orthostatic hypotension	5	2.0
Captopril + insulin	Increased risk of hypoglycemia	5	2.0
Atenolol + insulin	Glycemic alterations; reduced hypoglycemic symptoms	5	2.0
Ferrous sulfate + omeprazole	Reduced iron bioavailability	4	1.6
Ferrous sulfate + levothyroxine	Reduced levothyroxine serum levels	4	1.6
Levothyroxine + pantoprazole	Reduced effectiveness of levothyroxine	4	1.6
Atenolol + warfarin	Increased prothrombin time or INR	4	1.6

CNS = central nervous system; INR = international normalized ratio.

The most common moderate interaction was between insulin and losartan, which was observed in the drug therapy prescribed for 17 patients (6.7%). The most common moderate interactions identified in this study and their clinical consequences, as per DrugReax, are shown in Table 2.

Five types of contraindicated drug interactions were identified, from which the most frequent clinical effects were increased risk of QT prolonged interval (fluconazole plus salmeterol; and domperidone plus fluconazole), followed by increased risk of extrapyramidal reactions (bromopride plus venlafaxine) and increased risk of myopathy and rhabdomyolysis (ketoconazole plus simvastatin; and clarithromycin plus simvastatin).

Drug interactions that increase the risk of falls and fractures were the most frequent (n = 4) among those that should be avoided among older adults, as per the 2015 AGS/Beers criteria. Among the older people investigated here, three presented potential drug interactions between opioid receptor agonist analgesics and two or more drugs acting on the central nervous system (CNS). The potential drug interactions identified in this study, which should be avoided among older adults, as per the 2015 AGS/Beers criteria, are described in Table 3.

**Table 3.** Description of drug interactions that should be avoided among older adults, as per the 2015 American Geriatric Society (AGS)/Beers criteria, in the drug therapy prescribed for older adults at hospital discharge

Object drug and class	Interacting drug and class	Absolute frequency	Risk rationale
Benzodiazepines	≥ 2 medications that act on the CNS		Increased risk of falls and fractures
Clonazepam	Tramadol + quetiapine	1	
Opioid receptor agonist analgesic	≥ 2 medications that act on the CNS		Increased risk of falls
Tramadol	Clonazepam + sertraline	3	
Codeine	Quetiapine + sertraline		
Methadone	Haloperidol + lorazepam		
Peripheral alpha-1 blocker	Loop diuretics		Increased risk of urinary incontinence in women
Doxazosin	Furosemide	1	
	NSAIDs		Increased risk of bleeding
Warfarin	Nimesulide	1	

CNS = central nervous system; NSAIDs = nonsteroidal anti-inflammatory drugs.

Table 4 shows the results from the univariate and multivariate analyses on the factors associated with occurrences of drug interactions. In the univariate analyses, statistically significant associations were found between drug interactions and polypharmacy (P = 0.00) and between drug interactions and high drug therapy complexity index (P = 0.00). In the multivariate analysis, the factors that remained associated with drug interactions were polypharmacy (odds ratio, OR = 12.62; 95% confidence interval, CI 6.25-25.50; P = 0.00), diabetes mellitus (OR = 2.16; 95% CI 1.05-4.44; P = 0.04), hypothyroidism (OR = 7.29; 95% CI 2.03-26.10; P = 0.00), chronic kidney disease (OR = 3.41; 95% CI 1.09-10.64; P = 0.03) and hospitalization in geriatric units (OR = 0.45; 95% CI 0.22-0.89; P = 0.02).

**Table 4.** Univariate and multivariate analyses on factors associated with occurrences of drug interactions in the drug therapy prescribed for 255 older adults at hospital discharge

Description	Interactions		Univariate analysis		Multivariate analysis	
Variable	Frequency					
	Yes n (%)	No n (%)	Odds ratio (95% CI)	P-value	Odds ratio (95% CI)	P-value
Sociodemographic characteristics						
Gender						
Female	103 (59.5%)	43 (52.4%)	1.33 (0.79-2.26)	0.28		
Male	70 (40.5%)	39 (45.6%)	1			
Age						
≥75	91 (52.6%)	49 (59.8%)	0.75 (0.44-1.27)	0.28		
<75	82 (47.4%)	33 (40.2 %)	1			
Clinical characteristics						
Geriatric unit						
Yes	58 (33.5%)	39 (47.6%)	0.56 (0.32-0.95)	0.03^*^	0.45 (0.22-0.89)	0.02
No	115 (66.5%)	43 (52.4%)	1		1	
Stroke						
Yes	26 (15.0%)	11 (13.4%)	1.14 (0.53-2.44)	0.73		
No	147 (85.0%)	71 (86.6%)	1			
Heart failure						
Yes	35 (20.2%)	3 (3.7%)	6.68 (1.99-22.42)	0.00^*^		
No	138 (79.8%)	79 (96.3%)	1			
COPD						
Yes	25 (14.5%)	9 (11.0%)	1.37 (0.61-3.09)	0.45		
No	148 (85.5%)	73 (89.0%)	1			
Cancer						
Yes	18 (10.4%)	11 (13.4%)	0.75 (0.34-1.67)	0.48		
No	155 (89.6%)	71 (86.6%)	1			
Diabetes mellitus						
Yes	90 (52.0%)	20 (24.4%)	3.36 (1.87-6.04)	0.00^*^	2.16 (1.05-4.44)	0.04
No	83 (48.0%)	62 (75.6%)	1		1	
Pneumonia						
Yes	32 (18.5%)	21 (25.6%)	0.66 (0.35-1.23)	0.19^*^		
No	141 (81.5%)	61 (74.4%)	1			
Dementia						
Yes	50 (28.9%)	29 (35.4%)	0.74 (0.42-1.30)	0.30		
No	123 (71.1%)	53 (64.6%)	1			
Hypothyroidism						
Yes	39 (22.5%)	4 (4.9%)	5.67 (1.95-16.48)	0.00^*^	7.29 (2.03-26.10)	0.00
No	134 (77.5%)	78 (95.1%)	1		1	
Chronic kidney disease						
Yes	40 (23.1%)	5 (6.1%)	4.63 (1.75-12.23)	0.00^*^	3.41 (1.09-10.64)	0.03
No	133 (76.9%)	77 (93.9%)	1		1	
Peripheral vascular disease						
Yes	9 (5.2%)	7 (8.5%)	0.59 (0.21-1.64)	0.30		
No	164 (94.8%)	75 (91.5%)	1			
Asthma						
Yes	3 (1.7%)	0 (0.0%)	1.48 (1.36-1.61)	0.55^**^		
No	170 (98.3%)	82 (100.0%)	1			
Arterial hypertension						
Yes	131 (75.7%)	50 (61.0%)	1.99 (1.14-3.51)	0.01*		
No	42 (24.3%)	32 (39.0%)	1			
Acute myocardial infarction						
Yes	16 (9.2%)	3 (3.7%)	2.68 (0.76-9.48)	0.11^*^		
No	157 (90.8%)	79 (96.3%)	1			
Depression						
Yes	36 (20.8%)	10 (12.2%)	1.89 (0.89-4.03)	0.09		
No	137 (79.2%)	72 (87.8%)	1			
Functional characteristics						
VES-13 median						
≥ 5	90 (52.0%)	46 (56.1%)	0.85 (0.50-1.44)	0.54		
< 5	83 (48.0%)	36 (43.9%)	1			
Drug therapy-related characteristics						
Polypharmacy with 5 drugs						
Yes	149 (86.1%)	25 (30.5%)	14.15 (7.48-26.79)	0.00^*^	12.62 (6.25-25.50)	0.00
No	24 (13.9%)	57(69.5%)	1		1	
MRCI index						
Yes	117 (67.6%)	16 (19.5%)	8.62 (4.58-16.22)	0.00^*^		
No	56 (32.4%)	66 (80.5%)	1			

^*^P-value < 0.2: variable selected for the multivariate logistic regression; ^**^P-value was calculated using Fisher’s exact test; Hosmer-Lemeshow test: chi-square = 4.958; degrees of freedom = 7; P = 0.665.

CI = confidence interval; COPD = chronic obstructive pulmonary disease; VES-13 = Vulnerable Elders Survey-13; MRCI = Medication Regimen Complexity Index.

## DISCUSSION

This study showed that potential drug interactions occurred very frequently in the drug therapy that was prescribed for older adults at discharge from hospital. This result is compatible with the findings from previous studies involving older patients at hospital discharge, which also showed high prevalence of drug interactions in this setting.^17^^,^^18^ The number of drugs prescribed was significantly associated with the number of drug interactions. Patients with diabetes mellitus, hypothyroidism or chronic kidney disease (CKD) also had more drug interactions than did those with other comorbidities. The frequency of drug interactions was lower among the older adults who had been hospitalized in the geriatric unit.

High prevalence (54.5%) of major drug interactions was observed in this study. These can potentially increase the risks of cardiotoxicity, bleeding, rhabdomyolysis and hypoglycemia, which may compromise the functionality and quality of life of older people.^9^ As shown in previous studies, high frequency of potentially harmful drug interactions at hospital discharge was noted. Angiotensin-converting enzyme inhibitors (ACE inhibitors), diuretics, beta-blockers, antiplatelet agents, antidiabetics and oral anticoagulants were the drugs most involved in these interactions.^18^^,^^19^

Among the most common major drug interactions, the interaction between amlodipine and simvastatin was most prevalent. This drug interaction may increase the risks of rhabdomyolysis and myopathy.^20^^,^^21^ Combined therapies between statins and other cardiovascular medications are common, and the potential for significant drug interactions needs to be borne in mind, since such interactions increase the exposure to statins and, consequently, elevate the risk of myopathy.^20^^,^^21^^,^^22^

High prevalence of drug interactions with medications of the ACE inhibitor class was frequently observed in this study. Changes to renal function relating to aging increase the susceptibility of older adults to the nephrotoxic effects of ACE inhibitors, especially if administered with other medications. A prospective study in which the aim was to identify current drug-drug interaction (DDI) that resulted in adverse drug reactions among older patients within 30 days of discharge from an internal medicine clinic found that older adult patients who had been prescribed furosemide and ACE inhibitors presented asymptomatic elevated serum creatinine during the treatment and underwent dose adjustment of ACE inhibitors and diuretics.^18^

Studies on older patients receiving outpatient treatment have also identified high prevalence of concomitant use of ACE inhibitors and diuretics for blood pressure control.^23^^,^^24^ Despite the greater effectiveness of this combination of antihypertensives, the combination of these medications increases the risk of orthostatic hypotension, especially among older people, with a risk of injury due to falls.^25^

In a study conducted in the south of Brazil, the frequency with which patients used antidiabetics and antihypertensives was high,^26^ due to high prevalence of diabetes mellitus and arterial hypertension. While some interactions with antidiabetics are desirable and promote glycemic control in patients with diabetes, continuous blood glucose measurement should be performed to ensure that hypoglycemia does not occur in older adults.^26^ Hypoglycemic events among the elderly are associated with worsened cognitive decline and increased frequency of cardiovascular events. Furthermore, sarcopenia, peripheral neuropathy, dysautonomia and reduced visual acuity among older people elevate the risk and severity of falls, favored by episodic hypoglycemic events.^27^

Among the drugs for which concomitant prescriptions are contraindicated, those that had the clinical effect of increasing the risk of prolonged QT interval were observed more frequently. Among the risk factors for this event were female sex, aging and a combination of drugs that alone prolong the QT interval or increase the likelihood of this event through inhibiting drug metabolism. This prolongation may trigger the onset of ventricular arrhythmias and result in sudden death, which highlights the importance of avoiding drug interactions that expose older adults to higher risk of prolonging the QT interval and the importance of monitoring patients in cases in which such combinations are necessary.^25^

One innovative approach in the present study comprised identification of drug interactions that should be avoided among older people, as per the 2015 AGS/Beers criteria. The recommendations of these criteria stem from observation that these interactions are highly associated with clinically relevant adverse events among older adults.^11^

Associations that elevated the risk of falls were most frequent. Besides the clinical consequences, such as increased morbidity and mortality, falls among older people lead to social, economic and psychological harm, thereby increasing dependence and institutionalization.^28^ An ecological study on fall-related admission and mortality rates among older adults showed that the mortality and hospitalization rates due to falls were higher among older people in Brazil. This emphasizes that use of three or more drugs acting on the central nervous system should be avoided in the geriatric population.^29^

Occurrences of drug interactions were positively associated with the number of drugs used, which was in line with other published studies.^30^^,^^31^ A retrospective study in which the trends and determinants of polypharmacy among patients discharged from an internal medicine unit in a teaching hospital were measured showed that the risk factors for polypharmacy were age older than 75 years, high CCI and multiple morbidities.^17^ The presence of multiple morbidities in older adults favors prescription of several medications. Moreover, the widespread reality is that doctors do not have access to all the medicines that their patients are already using. This favors prescription of a more significant number of medications and highlights the importance of drug therapy reconciliation during the transition of care from the hospital setting to the patient’s home.^25^

In addition to polypharmacy, the multivariate analysis identified that the factors relating to higher frequencies of drug interactions were diabetes mellitus, hypothyroidism and CKD. Regarding glycemic control among patients with diabetes, prescription of oral antidiabetics such as metformin, with or without associated prescription of insulin, may be necessary to improve the effectiveness of treatment. This may favor drug interactions among older adults, since the present study showed that metformin and insulin are medications often associated with potential drug interactions. Also, diabetes in older people is usually accompanied by cardiovascular complications and other comorbidities.^27^^,^^32^ Therefore, in this population, use of five or more medications is frequent and enables drug interactions.^32^

Regarding hypothyroidism, it is known that thyroid-related diseases are highly prevalent and occur more frequently among older women.^33^ The treatment indicated for this condition is based on replacement doses of levothyroxine sodium, a medication for which bioavailability is modified by drug interactions with ferrous sulfate and proton pump inhibitors.^34^ This may explain the positive association between hypothyroidism and drug interactions among the older adults investigated here.

Furthermore, it was observed that the elderly individuals with CKD had higher numbers of drug interactions. Patients with this disease are at high risk of metabolic and cardiovascular complications and, consequently, require use of polypharmacy, which is one of the factors involved in occurrences of drug interactions.^35^

Lastly, it was observed that hospitalization in the geriatric unit gave rise to lower likelihood of drug interactions at hospital discharge. Older individuals have particular health characteristics and specific medical needs that are more likely to be taken into consideration by geriatricians, who have specialized knowledge and skills. Geriatricians have greater awareness of the risks of adverse drug events among older adults and, therefore, during hospitalization. They can streamline the drug therapy strategy, including deprescription of medications, which may contribute towards lower levels of interactions.^36^

Given the high frequency of drug interactions and polypharmacy, there is an evident need for an approach that ensures safe drug therapy for older adults at discharge from hospital. The strategies that might be involved include analysis of the dose used, consideration of possible therapeutic alternatives and patient monitoring, along with the structuring of a multidisciplinary team composed of a geriatrician, a clinical pharmacist and a nurse.^4^^,^^26^ Interventions conducted by pharmacists within this practical setting, such as drug therapy reviews and actions planned with the prescriber, may reduce the number of older patients with clinically-relevant drug interactions.^5^

The strength of the present study lay in its use of software with sensitivity and specificity that were suitable for identifying drug interactions among older adults. However, this study had significant limitations. First, the sample was non-probabilistic, which may have compromised the external validity of the results found. Second, the study was conducted in a hospital that is not part of the Brazilian National Health System (Sistema Único de Saúde, SUS) and thus may not reflect the reality of most older people in Brazil. Also, this study focused on potential drug interactions and did not identify interactions with clinical manifestations. Furthermore, the durations of treatments were not considered in identifying drug interactions. Therefore, the frequency of drug interactions may have been overestimated, since some of these interactions need mechanisms for enzyme induction and inhibition that are time-dependent.

## CONCLUSIONS

The frequency of potential drug interactions in the drug therapy prescribed to these older adults at discharge from hospital was high. Polypharmacy, diabetes mellitus, hypothyroidism and chronic kidney disease were positively associated with occurrences of potential drug interactions, and admission to a geriatric unit was inversely associated with such occurrences.
